# Implementing social and affective touch to enhance user experience in human-robot interaction

**DOI:** 10.3389/frobt.2024.1403679

**Published:** 2024-08-12

**Authors:** M. Ege Cansev, Alexandra J. Miller, Jeremy D. Brown, Philipp Beckerle

**Affiliations:** ^1^ Chair of Autonomous Systems and Mechatronics, Department of Electrical Engineering, Friedrich-Alexander-Universität Erlangen-Nürnberg, Erlangen, Germany; ^2^ Haptics and Medical Robotics Laboratory, Johns Hopkins University, Department of Mechanical Engineering, Baltimore, MD, United States; ^3^ Department of Artificial Intelligence in Biomedical Engineering, Friedrich-Alexander-Universität Erlangen-Nürnberg, Erlangen, Germany

**Keywords:** human-robot interaction, user experience, affective touch, trust, semi-autonomous systems, upper-limb prosthetics, haptic feedback

## Abstract

In this paper, we discuss the potential contribution of affective touch to the user experience and robot performance in human-robot interaction, with an in-depth look into upper-limb prosthesis use as a well-suited example. Research on providing haptic feedback in human-robot interaction has worked to relay discriminative information during functional activities of daily living, like grasping a cup of tea. However, this approach neglects to recognize the affective information our bodies give and receive during social activities of daily living, like shaking hands. The discussion covers the emotional dimensions of affective touch and its role in conveying distinct emotions. In this work, we provide a human needs-centered approach to human-robot interaction design and argue for an equal emphasis to be placed on providing affective haptic feedback channels to meet the social tactile needs and interactions of human agents. We suggest incorporating affective touch to enhance user experience when interacting with and through semi-autonomous systems such as prosthetic limbs, particularly in fostering trust. Real-time analysis of trust as a dynamic phenomenon can pave the way towards adaptive shared autonomy strategies and consequently enhance the acceptance of prosthetic limbs. Here we highlight certain feasibility considerations, emphasizing practical designs and multi-sensory approaches for the effective implementation of affective touch interfaces.

## 1 Introduction

Touch is vital for a human to live a healthy life (Field, 2014). According to Maslow’s Hierarchy of Needs, to lead a healthy life, one must have physiological health, safety and security, love and belonging, self-esteem, and self-actualization (Maslow, 1943). Many of these are emotional needs, and as sentient beings, we must account for our emotions in human-robot interaction. One way emotions are influenced is through affective and social touch (McGlone et al., 2014; Schirmer et al., 2023). The ideal human-robot interaction scenario should consider this and provide avenues for haptic communication.

In addition to claiming that human-human interactions benefit from the affective components of touch, we also envision that both the user and the robot can benefit from affective communication during human-robot interaction. On one hand, affective touch can trigger a wide range of emotional responses, including pleasantness through gentle touch (Essick et al., 1999), embodiment of artificial limbs (Crucianelli et al., 2013), and calming effects under stressful conditions (Morrison, 2016). On the other hand, certain psychological factors, such as trust and cognitive load, significantly shape the nature of interaction with a (semi-)autonomous system (Lee and See, 2004; Hancock et al., 2011). At this point, we seek to answer the question: “Can we propose a conceptual method to incorporate affective touch into human-robot interaction to improve the user experience and accordingly enhance the nature and efficiency of the interaction?”

In the unique case where the robot becomes a part of the human body, as in an upper-limb prosthesis, social touch is inherently intertwined in the interaction. Affective communication could be included to improve the experience of using an upper-limb prosthesis because our hands are more than just functional tools. They are mediums through which social connection occurs. Thus, people who wear prostheses and do not experience the same tactile sensations as those who do not, must be included in our research on social touch interactions. Despite significant improvements in prosthesis technology, 44% of wearers still reject their clinical devices (Salminger et al., 2022). At the same time, it has been shown that those wearers who keep their devices largely use them for non-prehensile (grasping) manipulations (Spiers et al., 2021). In addition to excessive prosthesis weight (Biddiss et al., 2007), improper socket fit (Daly et al., 2014), and unreliable function (Salminger et al., 2022), researchers have suggested that a lack of haptic feedback has contributed to prosthesis rejection and disuse (Niedernhuber et al., 2018; Stephens-Fripp et al., 2018). To remedy this, research has explored both invasive and non-invasive approaches to relaying discriminative haptic feedback to aid prosthesis users in functional task execution (Bensmaia et al., 2020; Sensinger and Dosen, 2020). For example, researchers have demonstrated that vibrotactile feedback of grip force combined with autonomous grasp control leads to improved task execution and lower mental effort (Thomas et al., 2023a; b). Likewise, researchers have demonstrated that invasive peripheral nerve feedback of grasp pressure leads to both functional and psychosocial improvements for prosthesis wearers (Chee et al., 2022). More recently, researchers have demonstrated that providing non-invasive thermal feedback to prosthesis wearers also provides functional utility (Iberite et al., 2023; Osborn et al., 2023). While these advances have significantly advanced our understanding of the importance of haptic feedback in dexterous task execution, our understanding of their utility in social interactions, in particular those involving affective touch are not well understood.

**FIGURE 1:**
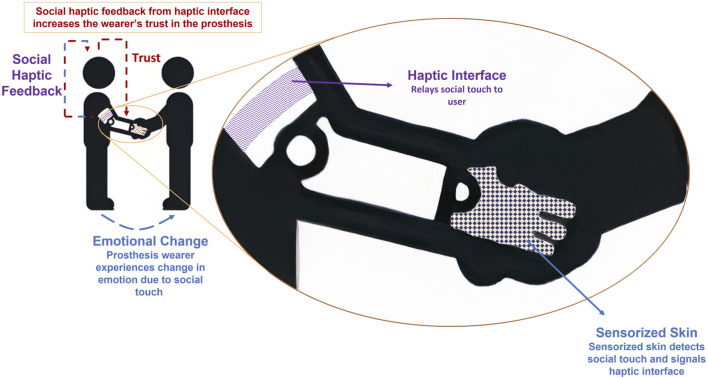
An overview of the presented perspectives exemplified in a handshake scenario involving an individual with a prosthetic arm. In this setup, the prosthetic hand is equipped with sensorized skin to detect social touch. The haptic interface, seamlessly integrated into the socket of the prosthetic arm, then transmits the detected touch to the residual limb of the prosthesis wearer. The wearer’s emotional state may undergo positive changes during social touch. Additionally, the haptic interface can autonomously mediate this touch, stepping in to restore trust when the wearer’s confidence in the prosthesis diminishes, in this or other appropriate scenarios. This approach holds the potential to significantly enhance both user experience and prosthesis performance, especially when integrated into shared autonomy strategies.

Figure 1 provides an overview of one of the potential applications of social touch in an exemplary prosthesis handshaking scenario. In later sections, we use the terms *social touch* and *affective touch*. While affective touch primarily aims to elicit emotional responses and foster bonding and comfort with a stronger emphasis on its neurophysiological background, social touch serves a broader range of social functions and conveys messages related to social interactions and relationships (Cascio et al., 2019). Given the critical roles both types of touch play in human communication, bonding, and wellbeing, existing literature approaches these concepts from distinct perspectives. We intentionally use both terms in their respective contexts to differentiate between broad social touch interactions, potentially experienced by prosthesis users, and affective touch that describes touch that stimulates CT afferents.

## 2 Affective touch, trust, and shared autonomy

Considering affective touch as a haptic communication channel, which has been previously explored primarily in its hedonic aspects, may contribute significantly to fostering trust between collaborating agents engaged even in tasks defined solely by functionality. In semi-autonomous systems, trust is a key psychological factor, influencing user interactions and task performance (Hancock et al., 2011). Hence, we inquire whether trust can serve as a link that improves interactions with semi-autonomous systems through the incorporation of affective touch. We posit that establishing such a connection during human-robot interaction is feasible, especially if wearable haptic interfaces, such as prosthetic arms, can autonomously convey affective touch or realistically transfer emotional cues from another individual.

### 2.1 The emotional impact of affective touch

Humans have multiple means to express their emotions, verbally through words and tone of voice or non-verbally through gestures, facial expressions and touch. Hertenstein et al. (2009) showed that eight distinct emotions, i.e., anger, fear, disgust, sadness, happiness, love, gratitude, and sympathy, can be communicated *via* touch. Although affective touch relates to the tactile communication of any emotional and social information (McGlone et al., 2014), pleasantness is one of the strongly elicited emotions that is linked to affective touch (Essick et al., 1999; Ackerley et al., 2014; Pawling et al., 2017). Due to this link, it is even mentioned as pleasant touch by Löken et al. (2009); Essick et al. (2010). Beyond being perceived as pleasant, affective touch enhanced perceived embodiment of a rubber hand (Crucianelli et al., 2013; Van Stralen et al., 2014) in variations of the classical rubber hand illusion experiment (Botvinick and Cohen, 1998). From a neurological perspective, Morrison (2016) claims that affective touch acts as a stress buffer, i.e., calibrates the stress responses of the body. Affective touch also reduces anxiety levels and autonomic responses, e.g., skin conductance and heart rate variability, which indicates a calming effect, when administered to a partner (Mazza et al., 2023). Therefore, including affective touch in human-robot interaction may be beneficial to the human’s experience.

### 2.2 Human-centered perspective on shared autonomy

Recently, semi-autonomous systems have been playing an important role in daily and professional life. They serve as a bridge towards fully autonomous systems, aiming to alleviate both physical and cognitive burdens on humans. Besides, semi-autonomous systems can be more favorable than autonomous systems in certain scenarios. Despite significant progress in machine learning and artificial intelligence, human expertise remains indispensable for complex tasks like surgical procedures involving medical robots, potentially enhancing patient trust Moustris et al. (2011). Moreover, human-in-the-loop systems offer flexibility and adaptability, crucial in dynamic and uncertain environments, such as semi-autonomous vehicles navigating through traffic congestion (Trautman et al., 2015; Schwarting et al., 2018).

Human-in-the-loop shared control approaches can also be useful in upper-limb prosthetics given the high dexterity required. Two main challenges in control of upper-limb prostheses are high cognitive burden of prosthesis use (Thomas et al., 2023b) and unnatural grasp movements that are incompatible with the motion of the intact limb (Guo et al., 2023). They reviewed various semi-autonomous control strategies that can reduce the workload of users and nonhuman behavior of prosthesis joints.

We claim that semi-autonomous systems, capable of adjusting their behavior while considering user experience, can enhance both performance and overall user satisfaction. Specifically, we propose the use of affective touch as a means to elevate the user experience during human-robot interaction. Achieving this goal requires a thorough understanding of the psychological factors influencing shared autonomy.

### 2.3 Psychological factors shaping shared autonomy

Enhancing the quality of interaction, whether it involves human-human, human-computer, or human-robot interaction, requires thoughtful consideration of the psychological states and needs of individuals involved (Cansev et al., 2021). The study of user experience (UX) in interactions with intelligent systems has gained attention in recent years (Shneiderman et al., 2017; Beckerle et al., 2018; Alenljung et al., 2019; Torricelli et al., 2020). The primary objective is to apprehend how technology can enhance the overall experience for its users. The subsequent challenge is considering the human factors as design criteria for interactive systems (Prati et al., 2021; Hao et al., 2022). By combining these approaches, developing human-centered designs will lead to enhanced UX, such as, enhanced embodiment of interactive systems (Schofield et al., 2021), which in turn fosters the research and advancements on human-centered designs including semi-autonomous systems.

Trust of a user on a semi-autonomous system is one of the most frequently considered psychological factors in discussions about shared autonomy. According to Lee and See (2004), trust is a multidimensional concept influenced by analytic, analogical, and affective interpretations of automated systems. They posit that emotions play a pivotal role, as affective processes significantly influence both analytic and analogical responses, emphasizing that trust is not only a cognitive consideration but also an emotional experience (Fine and Holyfield, 1996; Lee and See, 2004). At this point, we consider the potential impact of affective touch on eliciting emotional responses for trust-building in (semi-)autonomous systems while acknowledging the contribution of task-related analytic and analogical cognitive processes.

Analyzing and generalizing trust in automation is challenging as trust can fluctuate due to many human-related, robot-related, and environmental factors (Hancock et al., 2011; Schaefer et al., 2016). As shown in the review by Kohn et al. (2021), self-report measures along with behavioral measures are preferred over physiological measures (e.g., electrodermal activity, eye gaze tracking, and heart rate change and variability) in interpersonal trust analysis, because they are easy to validate and integrate into interaction. We believe incorporating physiological measures more often in trust analysis is important for avoiding biased results between subjective and objective measures as well as enabling real-time trust analysis. We agree with Hancock et al. (2011) that potential discrepancies between individual’s self-report and behavior can only be monitored when subjective measures are combined with objective measures. We posit that objective physiological measurements must be considered to accurately picture the state of trust. Additionally, we argue that real-time measurement of trust appears essential to create shared autonomy strategies that can adapt the behavior of semi-autonomous systems based on the trust of the user. To the best of our knowledge, the self-reported trust measurement with the most real-time capability assesses trust every 25 s *via* gamepad buttons (Desai et al., 2013). Such a measurement frequency would not suffice for responsive interaction in dynamically changing conditions. Among physiological measurements, both electrodermal activity and heart rate positively correlated with stress, anxiety, and cognitive workload (Caplan and Jones, 1975; Payne and Rick, 1986; Jacobs et al., 1994), emerge as valuable indicators of trust (Waytz et al., 2014; Akash et al., 2018). Since affective touch has soothing effects, e.g., acting as a stress buffer (Morrison, 2016) and reducing anxiety levels and autonomic responses under certain conditions (Mazza et al., 2023), as explained in Section 1, we believe that it can improve the trust of a person on a semi-autonomous system. For example, a prosthetic arm can autonomously mediate affective touch on the residual limb to soothe the wearer when trust on the prosthesis is decreased. This is why the real-time and accurate estimation of trust is important.

While trust is highlighted as the principal motivator and modulator of shared autonomy in this paper, we anticipate that agency, i.e., the feeling of being in control of an object’s movements (Braun et al., 2018), constitutes another psychological factor, aligning with the task-related analytic cognitive processes as defined by Lee and See (2004), in our perspective. Crucianelli et al. (2013) demonstrated that affective, slow stimuli not only enhances perceived ownership, but also improves the sense of agency, a sub-factor of embodiment (Schettler et al., 2019). The roles of other affect-related factors during semi-autonomous interaction is still to be investigated.

## 3 Social touch for upper-limb prosthetic users

We believe prosthesis wearers should experience the richness and benefits of affective touch during tactile interactions. Holding hands with a partner or spouse regulated neural and physiological responses to receiving a small electrical shock (Coan et al., 2006). Among couples who were separated by distance, a haptic bracelet that gave a squeeze on the wrist enhanced their feelings of social connection (van Hattum et al., 2022). Verbal and visual cues of affection could still be expressed, but the inclusion of touch made a significant difference in promoting emotional wellbeing. This section discusses the need to understand social interactions involving prostheses and the potential benefits of providing social haptic feedback to upper-limb prosthetics users.

### 3.1 Studying the behavioral impact of social touch: the Midas touch

Social touch can induce changes in human behavior to an extent measurable by researchers in “real-world” scenarios. These changes in behavior are referred to as the Midas Touch Effect. Brief, light touches on the hand and/or arm compared to no tactile contact during social exchanges resulted in people tipping more at a restaurant (Crusco and Wetzel, 1984), being more likely to return and lend money (Kleinke, 1977), and spending more time and money inside a store (Hornik, 2014). Social touch not only affects human behavior in an altruistic manner, but it also impacts one’s attitude toward and evaluation of the toucher or environment. Students who were touched on the hand by library clerks when returning the library cards rated the library personnel and facilities more favorably than students who did not receive a touch (Fisher et al., 1976). People who received a light touch from a store employee while shopping or from a waiter in a restaurant rated the environments as more friendly than those who were not (Hornik, 2014). Social touch increased people’s willingness to participate in mall interviews (Hornik and Ellis, 1988). In a classroom setting, students were given the opportunity to write the solution to a statistical problem on the board. During the exercise, the teacher briefly touched a handful of students on the forearm. Results showed that being touched correlated with increased volunteering among the students (Guéguen, 2004). Additionally, bus drivers were more inclined to give passengers a free ride when they were touched on the arm while being asked the question (Guéguen and Fischer-Lokou, 2003). Finally, researchers have shown that people are more willing to give strangers a free cigarette if they were touched together with the request (Joule and Guéguen, 2007). Social touch is understated but plays an important role in our decision making and social behavior.

Current clinical prosthetics provide no cutaneous haptic feedback to the wearer, thus reducing the tactile interaction to a visual or purely kinesthetic stimulus: the person will see the touch or feel the force *via* the prosthetic socket on the residual limb from the touch. This begs the question, “What happens when one’s sense of touch is missing, such as for an upper-limb prosthetic user? Will the Midas Touch Effect remain if a person receives a touch on a prosthetic limb?” We hypothesize that prosthesis wearers do not experience it in the same manner, but an emotional change may still occur. Therefore, we advocate for researchers to investigate the social haptic needs of upper-limb prosthetics wearers. This supports placing equal emphasis on improving social touch experiences for prosthetics users and can enhance prosthesis acceptance and improve quality of life.

### 3.2 Studying social touch in upper-limb prostheses

We suggest that researchers investigate if and to what extent social touch enhances upper-limb prosthetics user experience. This may include distributing questionnaires that ask what their social touch experiences are in everyday life. Furthermore, user-studies that explore prosthesis wearer reaction in social touch scenarios, both in a lab and real-world setting, could be conducted. The Trier Social Stress Test (Kirschbaum et al., 1993), and other laboratory psychological experimental paradigms (McCarthy and Elson, 2018) have been used for decades to uncover underlying emotional experiences. For upper-limb prosthetics, the Midas Touch paradigm provides an appropriate framework for this, as previous studies without prostheses have been able to obtain quantitative data describing the effects of social touch.

To relay affective touch to the user, appropriate hardware must be used such as touch sensors on the prosthesis that signal haptic feedback displays to provide the cue to the wearer. Researchers have developed sensory skins specifically for prosthetics (Chortos et al., 2016; Yang et al., 2019; Chen et al., 2021), and sensors for social touch mediation (Eid and Al Osman, 2016; Huisman, 2017). Haptics researchers have created haptic displays that can relay social touch to the wearer (Huisman et al., 2013; Casini et al., 2015; Culbertson et al., 2018), which could be modified for prosthesis users. Furthermore, thermal displays can be incorporated to provide the realistic warmth felt during skin-to-skin contact in social touch studies (Iberite et al., 2023; Muheim et al., 2024).

To address the emotional and social aspects of the multi-faceted field of human-robot interaction, especially in prosthetics research, we suggest taking a holistic human approach and setting design requirements based on human needs. Maslow’s hierarchy of human needs involves social and emotional wellbeing, which are met through social haptic interaction. Recognizing social and psychological needs of a person, in addition to the physical functional requirements needed to lead a healthy life, will shift human-robot interaction research from a narrow focus on device advancement to the overall promotion of human wellbeing.

## 4 Operational considerations

**FIGURE 2:**
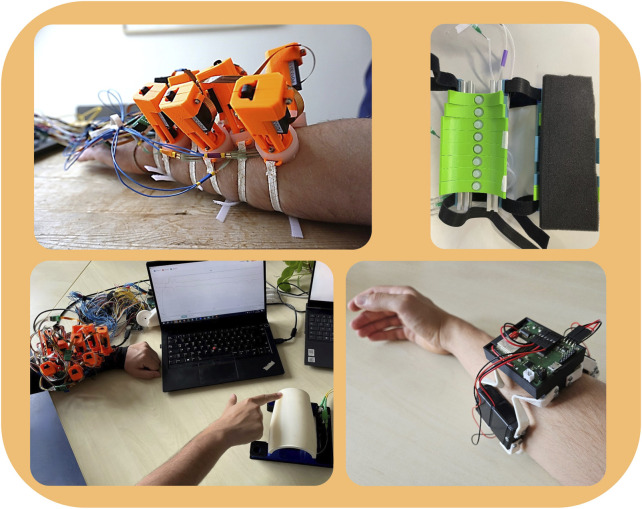
Examples of haptic interfaces for social affective touch applications. Top left: A 2D array of linear actuators creating indentations on the skin. Top right: A linear array of pneumatic stimulators. Bottom left: A design update on the array of linear actuators combined with sensorized skin. Bottom right: A vibrotactile bracelet with an inertial measurement unit for bi-directional haptic interaction.

Our perspectives are articulated primarily through insights derived from social affective touch. As shown in Figure 2, a variety of interfaces with different sensing and stimulation options, such as vibrotactile (Kindel et al., 2024), pneumatic (van Beek et al., 2023), and linear actuation (Ferguson et al., 2022) as well as tactile-sensitive skins (Massari et al., 2022), can be utilized to communicate social affective touch. Nonetheless, the current capabilities of interfaces designed to facilitate affective touch prompt the question of whether these interfaces can convincingly replicate human touch. The perception of affective touch may vary based on the chosen stimulation modality and conditions, with the possibility of it being perceived as unpleasant or even unsettling (Culbertson et al., 2018). Consequently, adherence to design guidelines is imperative to prevent any discomfort for prosthesis wearers. Additionally, the interfaces need to be wearable and sufficiently compact for seamless integration into the prosthesis socket.

Incorporating an additional haptic channel for affective interaction inevitably raises concerns about increased electronics, costs, design complexity, computational load, and further technical challenges due to the requirements of affective touch, potentially leading to performance degradation (Ege Cansev et al., 2021). We believe multisensory integration, which is described as the collaboration of sensory modalities and the integration of their informational content by Stein and Stanford (2008), *via* pseudo-haptic feedback, i.e., conveying haptic information just based on visual feedback (Lécuyer et al., 2000) can mitigate these potential issues. Pseudo-haptic feedback is used to represent multiple discriminative haptic properties, such as weight (Jauregui et al., 2014), stiffness (Lécuyer et al., 2001), friction and roughness (Costes et al., 2019). While implementing this solution in a virtual reality environment may not be suitable for scenarios such as a handshake *via* a prosthesis, the use of augmented reality to generate pseudo-haptic feedback can, at least partially, facilitate the communication of functional haptic information. This approach allows for the conservation of haptic resources, ensuring their availability for affective haptic exchanges.

## 5 Conclusion

We explored how affective touch can enrich the user experience in human-robot interaction, particularly focusing on upper-limb prosthetics and the impact of social touch. We advocate for further research into the broader implications of social touch in human-robot interaction and the potential benefits of integrating affective communication channels. By evoking positive emotions, affective touch can enhance user experience and foster trust, thereby improving collaborative tasks. Affective touch holds promise for influencing trust in shared autonomy scenarios, urging researchers to delve into how individuals using upper-limb prosthetics perceive affective touch and its effects on device usage. Wearable haptic interfaces capable of autonomously conveying affective touch could revolutionize human-robot interaction, facilitating more meaningful and emotionally connected interactions.

## Data Availability

The original contributions presented in the study are included in the article/supplementary material, further inquiries can be directed to the corresponding author.
